# Dysphoric symptoms in relation to other behavioral and psychological symptoms of dementia, among elderly in nursing homes

**DOI:** 10.1186/s12877-017-0603-4

**Published:** 2017-09-07

**Authors:** Agnes Lindbo, Maria Gustafsson, Ulf Isaksson, Per-Olof Sandman, Hugo Lövheim

**Affiliations:** 1 Department of Community Medicine and Rehabilitation, Geriatric Medicine, Umeå University, 901 87 Umeå, Sweden; 2 Department of Pharmacology and Clinical Neuroscience, Division of Clinical Pharmacology, Umeå University, 901 87 Umeå, Sweden; 3 Department of Nursing, Umeå University, 901 87 Umeå, Sweden; 4 Arctic Research Centre at Umeå University, 901 87 Umeå, Sweden; 5 Department of Neurobiology, Care Sciences and Society, Division of Nursing, Karolinska Institutet, Stockholm, Sweden; 6 Department of Health Sciences, University of Technology, Luleå, Sweden

**Keywords:** BPSD, Dementia, Cognitive impairment, Dysphoria, Depression, Apathy, Hallucination, Aggressiveness

## Abstract

**Background:**

Behavioral and psychological symptoms of dementia (BPSD) are common and varied in the elderly. The aim of the current study was to explore associations between BPSD and dysphoric symptoms at different levels of cognitive impairment.

**Methods:**

Assessments of 4397 elderly individuals living in nursing homes in Sweden were performed. Data on cognitive function and BPSD were collected using the Multi-Dimensional Dementia Assessment Scale (MDDAS). The relationships between dysphoria and eight BPSD factors were plotted against cognitive function to investigate how dysphoria affects BPSD throughout the dementia disease.

**Results:**

Overall, dysphoric symptoms were most prevalent in persons with moderate cognitive impairment. However, moderate to severe dysphoric symptoms showed no clear variation with cognitive impairment. Furthermore, aggressive behavior, verbally disruptive/attention-seeking behavior, hallucinatory symptoms and wandering behavior were more common with concurrent dysphoria regardless of cognitive function. In contrast, passiveness was more common with concurrent dysphoria in mild cognitive impairment but not in moderate to severe cognitive impairment.

**Conclusions:**

BPSD, including aggressive behavior and hallucinations, were more common with concurrent dysphoric symptoms, providing insight into behavioral and psychological symptoms among individuals with cognitive impairment. Apathy was more commonly associated with concurrent dysphoria at early stages of cognitive decline but not at later stages, indicating that apathy and dysphoria represent separate syndromes among elderly patients with moderate to severe cognitive impairment.

**Electronic supplementary material:**

The online version of this article (10.1186/s12877-017-0603-4) contains supplementary material, which is available to authorized users.

## Background

Dementia affects more than 35 million people worldwide [1]. In addition to cognitive decline, many people with dementia experience behavioral and psychological symptoms related to dementia (BPSD). More than 80% of people with dementia living in nursing homes have some form of BPSD [2]. These behavioral and psychological symptoms increase care giver burden [3], increase suffering and decrease quality of life for both the person with dementia and their caregiver [4].

The most common symptoms of BPSD are apathy (36%), symptoms of depression (32%) and agitation/aggression (30%) [5]. Prior research has focused on grouping BPSD into sub-syndromes that share the same etiology and perhaps treatment options. A review of 62 studies proposed the following grouping: hyperactivity (aggression and irritability), psychosis (delusions and hallucinations), mood disturbances (depression and anxiety) and euphoria. Apathy, eating disturbances, night-time behavior disturbances, disinhibition and aberrant motor behavior showed inconsistent results and thus did not fit into any grouping [6]. Whether mood disturbance and apathy are the same or separate syndromes has been the focus of some debate [7].

Depression is a form of BPSD that can be difficult to recognize. Studies using the same diagnostic criteria have reported a range of 19% to 40.5% for the prevalence of depression in nursing homes [8–10]. Furthermore, the reported prevalence of depression in Alzheimer’s disease has ranged from 5% to 44% depending on the diagnostic criteria used [11]. The large variability in reported prevalence reflects the difficulty of defining depression in dementia.

Previous studies suggest that some forms of BPSD are more likely to coincide with depression. In one study, 84.2% of people with dementia who also suffered from depression had other forms of BPSD compared to 50.0% of those without depression [10]. Another study reported that all BPSD were significantly more prevalent in dementia when depression was also present; large differences between those with and without depression were found in the symptoms of agitation, anxiety, irritability and hallucinations [12]. Furthermore, several studies have shown a strong association between both verbal and physical aggressive behavior and depression in the elderly [13].

There are somewhat conflicting results on how BPSD changes through the course of dementia. One prior study found that apathy increases almost linearly with severity of cognitive impairment, whereas other components of BPSD show higher prevalence rates in the middle stages of dementia [14]. Another study found the same positive correlation between apathy and cognitive impairment but no correlation between the prevalence of other BPSD and level of cognitive function in Alzheimer’s patients [15]. A prospective cohort study reported an increase in apathy severity and a decrease in depression severity with progression of dementia among nursing home residents [16, 17]. However, a review article examining 24 studies on Alzheimer’s disease found no clear association between dementia severity and prevalence of depression [18].

There is considerable overlap in symptoms in depression and dementia which leads to difficulty in the assessment of depression in severe dementia [19]. For example, apathy, or lack of interest, is a necessary criterion in diagnosing depression among cognitively intact individuals. However, apathy in severe dementia is not necessarily an emotional response but can be due instead to executive dysfunction, loss of concentration, psychomotor retardation or other cognitive deficits caused by neurodegeneration [19]. Thus, depression in dementia is ambiguous. Scales such as the Neuropsychiatric Inventory assess dysphoria, or depressed mood, characterized by sadness, guilt and hopelessness to define BPSD [20]. The current study uses measures of dysphoria instead of depression to investigate neuropsychiatric symptoms associated with dementia.

A more complete understanding of patterns and etiology of neuropsychiatric symptoms in dementia is vital for adequate clinical patient management. To our knowledge, no prior study has investigated BPSD with and without dysphoria and how the relationship changes through the course of dementia. Therefore, our aim was to explore associations between behavioral and psychological symptoms and dysphoria in relation to cognitive impairment.

## Methods

### Surveys and participants

The Multi-Dimensional Dementia Assessment Scale (MDDAS) [21] was administered at all nursing homes, including specialized care units for people with dementia, in Västerbotten County in northern Sweden, in 2007 and 2013. In addition, a small number of participants (*n* = 87) from the 2007 survey resided in hospital geriatric wards. Data from 2007 and 2013 were grouped together for statistical power. The surveys from both years used the same questionnaire and methodology.

The number of residents in the surveyed nursing homes was 3578 in 2007 and 3210 in 2013. The response rate was 85.8% in 2007 and 70.5% in 2013, resulting in participation of 3070 and 2262 individuals respectively. Those younger than 65 years (or for whom no information of age was registered) were excluded from the study. Those who were not rated on cognitive function and dysphoric symptoms were also excluded. The total number of people selected for participation was 2574 from the 2007 survey and 1823 from the 2013 survey, resulting in a total inclusion of 4397 individuals.

### Procedures

The MDDAS was sent to all nursing homes along with written instructions about how to carry out the assessments. A general description of the aim of the survey was provided in the instructions. Identical surveys were distributed to all the different nursing homes.

The MDDAS consists of easily observable behaviors and symptoms and does not require specific education to be understood.

A member of the research team was accessible by telephone to answer questions about the survey. The member of staff who knew each individual resident best was asked to fill in the assessment scale regardless of profession and education. The staff was informed that their assessment should be based on observations made during the preceding week.

The MDDAS rates function in daily life (ADL), cognition and behavioral and psychological symptoms [21]. The MDDAS has shown good intra- and inter-rater reliability [22]. ADL score was calculated based on the resident’s ability to cope with dressing, hygiene, eating, bladder control and bowel control. All ADL items were scored on a 5-point scale except for bladder control, which was scored on a 4-point scale. Total ADL scores therefore, varied from four to 24, where a higher score indicated greater ADL independence. Cognitive impairment was measured using Gottfries’ cognitive scale [23]. The scale consists of 27 items related to orientation and awareness. The maximum score is 27 points. A score of zero indicates severe cognitive impairment while a score of 24 points and above indicates no cognitive impairment. The cut-off at 24 points correlates with a sensitivity of 90% and a specificity of 91% to a score of 24 out of 30 on the Mini Mental State Examination (MMSE) [24]. Gottfries’ cognitive scale has been used since 1975 [23]. Over the years the scale has proven to be reliable and to have less of a floor effect than the MMSE [25].

The MDDAS assesses 25 behavioral and 14 psychological symptom items. Each symptom is rated on a 3-point scale that reflects frequency of the symptom: daily, at least one time/week or never during the observation time. The scores were then dichotomized to either at least once a week or less than once a week. Based on a factor analysis of the data from 2007, as previously published [26], the items were merged into 9 factors: aggressive behavior; regressive behavior; wandering behavior; passiveness; verbally disruptive/attention seeking behavior; restless behavior; hallucinatory symptoms; disoriented symptoms; and depressive symptoms. For each BPSD item there was up to 2.3% data missing and the ratings from these individuals were not included in the analysis of that specific BPSD item.

The survey contained a separate question for dysphoric symptoms that were rated on a 4-point scale: no dysphoric symptoms; mild, “somewhat sad but possible to divert sadness”; moderate, “seems blue and gloomy but may be diverted for short periods of time”; severe, “heavily blue and gloomy, cannot be diverted”. The last two ratings were grouped together since severe dysphoric symptoms were only present in 2.4% of participants. Therefore, there were three levels of dysphoria; no dysphoric symptoms, mild dysphoric symptoms and moderate to severe dysphoric symptoms.

### Statistics and calculations

As described above, based on a factor analysis, all 25 behavioral and 14 psychological items were merged into 9 BPSD factors (Additional file 1) [26]. The 39 contributing items had different bearing according to factor loading (correlation between BPSD factor and item), and different BPSD factors comprised different numbers of contributing items. For each BPSD factor therefore, a weighted score was calculated. This weighted score ranged from zero (no contributing symptoms present) to one (all contributing symptoms present), where the contributing items were weighted according to their factor loading and the total number of contributing items to the particular BPSD factor. An example is given for the BPSD factor hallucinatory symptoms. The factor analysis showed that the three contributing items “hallucinates visually” (factor loading 0.87), “hallucinates auditorially” (factor loading 0.87) and “talks to herself/himself” (factor loading 0.43) grouped together under the factor “hallucinatory symptoms” (Additional file 1) [26]. The weighted score for a participant with only visual hallucinations would be (1*0.87 + 0*0.87 + 0*0.43)/(0.87 + 0.87 + 0.43) = 0.4. For a person with all three symptom items present, the weighted score would be (1*0.87 + 1*0.87 + 1*0.43)/(0.87 + 0.87 + 0.43) = 1, and accordingly, for a person with no symptom item present, the weighted score would be (0*0.87 + 0*0.87 + 0*0.43)/(0.87 + 0.87 + 0.43) = 0. This procedure was performed for each BPSD factor. Then, for each BPSD factor, all weighted scores were summarized for all participants with the same score on Gottfries’ cognitive scale and the same level of dysphoria, and divided by the number of participants respectively, resulting in an mean weighted score for each BPSD symptom. The mean weighted score of each BPSD symptom were plotted in relation to cognitive function, separated for the three different levels of dysphoric symptoms.

Further, polynomial regression lines were fitted to the data. First-, second- and third-degree terms were entered into a multivariate linear regression for each factor. Significant coefficients (*p* < 0.05) were used in the final regression model. These results are shown as curves in the figures, with the different curves representing the different dysphoric levels.

An additional analysis was performed to compare the mean weighted score of each BPSD factor among participants with and without dysphoric symptoms in four different levels of cognitive function; (no (24-27 points on Gottfries’ cognitive scale), mild (16-23 points), moderate (8-15 points) and severe cognitive function (0-7 points)). In this analysis the three levels of dysphoria were dichotomized to no dysphoric symptoms, and dysphoric symptoms (mild dysphoric symptoms and moderate to severe dysphoric symptoms).

An independent-samples *t*-test was performed between the mean value of each BPSD factor with and without dysphoric symptoms. A Bonferroni’s correction was used and a *p*-value of less than 0.0016 was considered significant.

In the MDDAS, the symptom items “sad”, “crying”, “anxious and fearful”, were clustered together as depressive symptoms. To investigate concurrent validity of our separate rating of dysphoria we calculated the mean weighted score of the BPSD factor “depressive symptoms” at the three levels of dysphoric symptoms. The result was 0.103, 0.488 and 0.680 respectively in the groups “no dysphoria”, “mild dysphoria” and “moderate/severe dysphoria”, thus, strengthening the validity of the separate rating of dysphoria.

All data processing and statistical calculation were performed using the Statistical Package for Social Science SPSS^®^ version 22.0 and Microsoft^®^ Excel^®^ 2013.

## Results

**Fig. 1:**
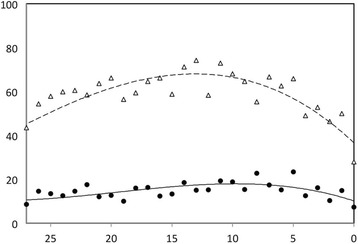
The prevalence of dysphoric symptoms in relation to level of cognitive function. The x-axis = score on Gottfries’ cognitive scale. The y-axis = percentage (%) of study population. Polynomial regression curves are fitted to the data. ∆ = all levels of dysphoric symptoms (mild, moderate and severe).● = moderate/severe dysphoric symptoms

The population characteristics are described in Table 1. The highest prevalence of dysphoric symptoms (74.2%) was registered at 13 points on Gottfries’ cognitive scale. The lowest prevalence (27.9%) was registered at zero on Gottfries’ cognitive scale (Fig. 1). The prevalence of moderate to severe dysphoria in relation to cognitive level varied from 7.4% to 23.3%, with no clear relationship to level of cognitive function (Fig. 1).

**Table 1:** Characteristics of study population

Total number of participants	4397
Female, n (%)	2972 (67.6)
Male, n (%)	1409 (32.0)
Mean age, years ± SD	84.7 ± 6.9
No cognitive impairment, n (%)	1332 (30.3)
Mild cognitive impairment, n (%)	1104 (25.1)
Moderate cognitive impairment, n (%)	1087 (24.7)
Severe cognitive impairment, n (%)	874 (19.9)
Dysphoric symptoms, n (%)	2490 (56.6)
Mild dysphoria, n (%)	1885 (42.9)
Moderate dysphoria, n (%)	499 (11.3)
Severe dysphoria n (%)	106 (2.4)
ADL score ± SD	15.6 ± 6.3

*ADL* activities of Daily Living, *SD* standard deviation

**Fig. 2:**
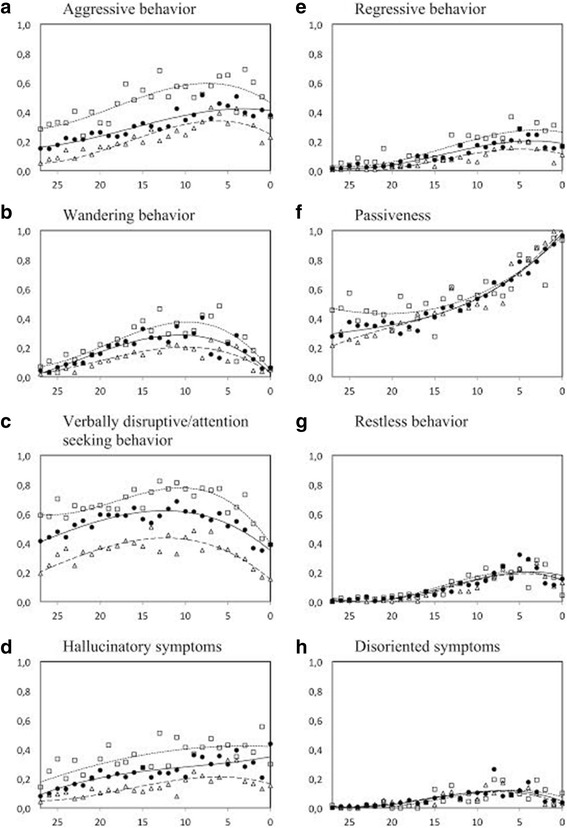
Behavioral and psychological symptoms in relation to level of cognitive impairment, for individuals with different levels of dysphoric symptoms. The x-axis = score on Gottfries’ cognitive scale. The y-axis = BPSD factor (the mean weighted score of contributing items- see text for further definition). Polynomial regression curves are fitted to the data. □ = severe to moderate dysphoric symptoms. ● = mild dysphoric symptoms. ∆ = no dysphoric symptoms. **a** Aggressive behavior. **b** Wandering behavior. **c** Verbally disruptive/attention seeking behavior. **d** Hallucinatory symptoms. **e** Regressive behavior. **f** Passiveness. **g** Restless behavior. **h** Disoriented symptoms

The mean weighted score of each BPSD factor, at varying severity of dysphoria, was plotted in relation to cognitive level (Fig. 2). The polynomial regression showed a significant relationship between cognitive score and BPSD within each dysphoric level (data not shown). Regression curves are included in the figures.

A comparison of groups with and without dysphoric symptoms in the four groups of cognitive function is presented in Table 2.

**Table 2:** Rating of BPSD factors, mean (± SD) in groups with and without dysphoric symptoms at different levels of cognitive impairment

BPSD item	Non-dysphoric	Dysphoric	P-value
Aggressive behavior			
27-24^a^	0.062 (0.155)	0.192 (0.228)	<0.001
23-16^a^	0.157 (0.234)	0.284 (0.284)	<0.001
15-8^a^	0.258 (0.307)	0.404 (0.328)	<0.001
7-0^a^	0.321 (0.334)	0.460 (0.358)	<0.001
Regressive behavior			
27-24^a^	0.007 (0.043)	0.019 (0.075)	0.002
23-16^a^	0.029 (0.097)	0.053 (0.125)	0.001
15-8^a^	0.083 (0.145)	0.128 (0.209)	<0.001
7-0^a^	0.146 (0.217)	0.203 (0.242)	0.001
Wandering behavior			
27-24^a^	0.026 (0.074)	0.055 (0.122)	<0.001
23-16^a^	0.107 (0.172)	0.185 (0.246)	<0.001
15-8^a^	0.190 (0.248)	0.313 (0.315)	<0.001
7-0^a^	0.114 (0.218)	0.186 (0.266)	<0.001
Passiveness			
27-24^a^	0.228 (0.295)	0.332 (0.324)	<0.001
23-16^a^	0.352 (0.331)	0.376 (0.328)	0.247
15-8^a^	0.500 (0.335)	0.508 (0.330)	0.693
7-0^a^	0.838 (0.256)	0.763 (0.281)	<0.001
Verbally attention seeking/disruptive behavior			
27-24^a^	0.219 (0.235)	0.464 (0.286)	<0.001
23-16^a^	0.354 (0.269)	0.597 (0.279)	<0.001
15-8^a^	0.412 (0.284)	0.645 (0.273)	<0.001
7-0^a^	0.276 (0.269)	0.536 (0.322)	<0.001
Hallucinatory symptoms			
27-24^a^	0,055 (0,168)	0.115 (0.241)	<0.001
23-16^a^	0,108 (0,226)	0.234 (0.340)	<0.001
15-8^a^	0,164 (0,280)	0.293 (0.354)	<0.001
7-0^a^	0,200 (0,293)	0.349 (0.389)	<0.001
Disoriented symptoms			
27-24^a^	0.004 (0.034)	0.005 (0.041)	0.053
23-16^a^	0.038 (0.127)	0.026 (0.096)	0.092
15-8^a^	0.084 (0.197)	0.098 (0.207)	0.301
7-0^a^	0.077 (0.208)	0.097 (0.216)	0.172
Restless behavior			
27-24^a^	0.003 (0.028)	0.009 (0.044)	0.009
23-16^a^	0.014 (0.058)	0.035 (0.094)	<0.001
15-8^a^	0.103 (0.169)	0.116 (0.185)	0.287
7-0^a^	0.189 (0.285)	0.210 (0**.**261)	0.259

*SD* Standard deviation, ^a^points on Gottfries’ cognitive scale

Aggressive behavior, wandering behavior, verbally disruptive/attention-seeking behavior and hallucinatory symptoms were more common with concurrent dysphoric symptoms at all stages of cognitive functioning (*p* < 0.001). Regressive behavior was more common with concurrent dysphoric symptoms in participants with reduced cognitive functioning (cognitive score of 0-23, *p* ≤ 0.001) but not in the group with normal cognitive functioning (cognitive score ≥ 24). Passiveness was more common with concurrent dysphoric symptoms at a normal cognitive functioning score (*p* < 0.001) compared to without dysphoric symptoms. At mild and moderate cognitive impairment no difference was seen between the groups with and without dysphoric symptoms. At moderate/severe cognitive impairment, passiveness was less common with concurrent dysphoric symptoms as compared to without dysphoric symptoms (*p* < 0.001). Restless behavior was generally uncommon among those surveyed. However, restlessness was more common with concurrent dysphoric symptoms in the group with mild cognitive impairment (p < 0.001). There was no difference in the presence of disoriented symptoms between groups with and without dysphoria.

## Discussion

The current study explored the relationship between behavioral and psychological symptoms and dysphoria in relation to level of cognitive impairment. Aggressive behavior, verbally disruptive/attention-seeking behavior, hallucinatory symptoms and wandering behavior were more common with concurrent dysphoria regardless of cognitive function. In contrast, passiveness was more common with concurrent dysphoria among those without cognitive impairment but not among those with cognitive impairment. Regressive behavior, restless behavior and disoriented symptoms were not correlated with level of dysphoria.

While the overall prevalence of dysphoric symptoms (50.6%) found in the present study was higher than the prevalence of depression (19-40.5%) previously reported [8–10], the prevalence of moderate to severe dysphoric symptoms found in the current study (13.8%) was lower. The difference likely reflects the difference in measurement instruments used. Dysphoric symptoms were most prevalent among participants with moderate cognitive impairment and less prevalent in cognitively intact participants and those with severe cognitive impairment. This finding is in agreement with previous studies [14, 16]. A drop in dysphoria rates in severe cognitive impairment may be due to structural changes in the brain. However, it is also possible that the presence of dysphoria and depression is underestimated in individuals with severe cognitive impairment due to a decreased ability to verbally and non-verbally communicate emotions. The prevalence of moderate to severe dysphoric symptoms was not correlated with cognitive level. A similar result was reported in a study of depression in relationship to severity of Alzheimer’s disease [18]. The current findings may indicate, in part, different causes of mild dysphoric symptoms and moderate to severe dysphoric symptoms among elderly with cognitive impairment.

Analysis of the current data found that aggressive behavior, wandering behavior, verbally disruptive/attention-seeking behavior and hallucinatory symptoms were more common with concurrent dysphoric symptoms at all levels of cognitive impairment. These results are in agreement with previous studies that have found an association between agitation and hallucination in patients with dementia [12, 13].

The association between agitation (including verbally disruptive behavior, aggressive behavior and wandering behavior) and dysphoric symptoms among persons with cognitive impairment may be explained by an imbalance in the monoamine system. Prior research has found a significant disruption in global serotonergic transmission in patients with dementia [27]. For example, a positron emission tomography study showed that the density of 5H_1A_ receptors was reduced in the right medial temporal cortex in patients with Alzheimer’s disease [28]. Furthermore, the density of hippocampal 5HT_1A_ receptors was significantly reduced in patients with Alzheimer’s disease and depression compared to Alzheimer’s patients without depression [29]. Moreover, reduced 5HT_1A_ receptor binding in the temporal cortex has been shown to correlate with aggressive behavior in Alzheimer’s disease [30]. Our results suggest an association between dysphoria and aggressive behavior in dementia and based on previous studies, it is possible that altered serotonergic transmission is a common denominator [27].

Apathy (passiveness) was more common with concurrent dysphoria among those without cognitive impairment but not among those with cognitive decline. This result is in agreement with longitudinal studies that have reported an increase in apathy but not depression as dementia progresses [16, 17]. These findings indicate that apathy and dysphoria are independent syndromes among people with moderate to severe cognitive impairment.

To our knowledge, the present study is the largest study to investigate the relationship between dysphoria and BPSD at different levels of cognitive impairment to date. The simplicity of the questions in the proxy-rated MDDAS based survey contributed to the validity of our results. On the other hand, the depression assessment used in this study is not a validated assessment, which would have been desirable. Also, that the survey was answered by staff members and not by the residents themselves, means that this assessment is an estimation and a possible source of errors. Also, we cannot be sure of how the staff have interpreted the questions asked in the survey, leading to possibly misclassification and confounding bias. Further, this study was a one-week cross-sectional study; meaning the data are point estimates and therefore reflect only the symptoms the person exhibited during that time. The symptoms might be affected by factors not corrected for, such as pharmacological treatment. One major limitation of this study is also the lack of data on diagnoses, including dementia diagnoses, which could act as a confounding variable in our study. In particular, visual hallucinations and depression are known to frequently occur in Parkinson’s disease and Lewy body dementia [31]. In this study, only people in nursing homes was assessed and the results should therefore not be generalised to other populations. All these limitations need to be taken into account when interpreting the results.

## Conclusion

While moderate to severe dysphoric symptoms do not vary with cognitive level, overall dysphoric symptoms are most prevalent at a moderate cognitive impairment. The discrepancy could indicate a difference in nosology. Aggressive behavior, wandering behavior, verbally disruptive/attention seeking behavior and hallucinatory symptoms were more common with concurrent dysphoric symptoms. Passiveness was more common with concurrent dysphoria at early stages of cognitive decline but not at later stages, supporting the notion that apathy and depression should be regarded as separate neuropsychiatric syndromes in individuals with dementia.

## Additional file

Additional file 1: Table S1. Factor analysis behavioral and psychological symptoms. (DOCX 20 kb)

## Abbreviations

ADL: Activities of Daily Living
BPSD: Behavioral and psychological symptoms of dementia
MDDAS: Multi-Dimensional Dementia Assessment Scale
MMSE: Mini Mental State Examination
SD: Standard deviation
SPSS: Statistical Package for Social Science
