# Supplementary material for: Dysphoric symptoms in relation to other behavioral and psychological symptoms of dementia, among elderly in nursing homes
Source: BMC Geriatr. 2017 Sep 7;17:206. doi: 10.1186/s12877-017-0603-4 (PMC5590234; doi:10.1186/s12877-017-0603-4)
Supplement: Additional file 1: Table S1. — Factor analysis behavioral and psychological symptoms. (DOCX 20 kb) [file 12877_2017_603_MOESM1_ESM.docx]

**Additional file 1: Table S1.** Factor analysis behavioral and psychological symptoms

| **Behavioral and psychological symptom item** | **Factor loading** | **Variance explained (%)** |
| --- | --- | --- |
| **Wandering behavior** |  | 7.56 |
| Packs up his/her things, is often on the way home | 0.76 |  |
| Often stands at the outer door wanting to go out | 0.69 |  |
| Hides things | 0.63 |  |
| Wanders back and forth alone or with other patients | 0.59 |  |
| Does not want to go to bed | 0.41 |  |
| Overactive/ manic | 0.34 |  |
| **Aggressive behavior** |  | 6.82 |
| Aggressive threats (words or gestures) to patients/staff | 0.82 |  |
| Easily annoyed | 0.75 |  |
| Hits patients/staff | 0.64 |  |
| Resists being dressed and undressed | 0.48 |  |
| Suspicious | 0.43 |  |
| **Verbally disruptive/ attention-seeking behavior** |  | 6.70 |
| Constantly seeks attention of the staff | 0.70 |  |
| Seeks help | 0.66 |  |
| Disturbed and restless | 0.57 |  |
| Interrupted night-time sleep | 0.51 |  |
| Shrieks and shouts continuously | 0.46 |  |
| Complains | 0.39 |  |
| **Restless behavior** |  | 6.24 |
| Rolls up tablecloths | 0.70 |  |
| Tears up newspaper, etc. | 0.67 |  |
| Mixes up food | 0.60 |  |
| Eats others’ food | 0.59 |  |
| Eats soil from potted plants, cigarette ends, etc. | 0.52 |  |
| **Regressive/ inappropriate behavior** |  | 5.64 |
| Unruly in bed, throws bedclothes on the floor | 0.61 |  |
| Smears faeces on clothes, furniture, etc. | 0.51 |  |
| Undresses in the dayroom | 0.64 |  |
| Piles up chairs, pushes tables, upends furniture | 0.47 |  |
| Spits out drugs | 0.36 |  |
| **Passiveness** |  | 5.45 |
| Lacks initiative | 0.72 |  |
| Does not talk spontaneously with patients/staff | 0.71 |  |
| Does not cooperate | 0.69 |  |
| **Depressive symptoms** |  | 5.27 |
| Sad | 0.83 |  |
| Crying | 0.81 |  |
| Anxious and fearful | 0.56 |  |
| **Hallucinatory symptoms** |  | 4.93 |
| Hallucinates visually | 0.87 |  |
| Hallucinates auditorially | 0.87 |  |
| Talks to her/himself | 0.43 |  |
| **Disoriented symptoms** |  | 4.71 |
| Lies in other patients’ beds | 0.70 |  |
| Urinates in wastepaper baskets, wash-basins or on the floor | 0.56 |  |
| Take things from other patients’ boxes and closets | 0.48 |  |
| **Cumulative variance explained** |  | 53.31 |

*Note.* A factor analysis (principal component analysis) with Varimax rotation was performed extracting factors with an eigenvalue of 1 or above. Items are presented in the bable under the factor to which they loaded the most. Published in: Gustafsson M, Sandman PO, Karlsson S, Gustafson Y, Lovheim H. Association between behavioral and psychological symptoms and psychotropic drug use among old people with cognitive impairment living in geriatric care settings. Int Psychogeriatr. 2013;25:1415-1423.
